# A Sad Occurrence—The Murder of Two Dentists

**Published:** 1875-08

**Authors:** 


*A Sad Occurrence.—The Murder of Two Dentists.*—The following sad occurrence is described by a Cynthiana, Ky., correspondent, and is an evidence of what the utterance of a single word may lead to :

Dr. E. J. Peckover and Dr. C. L. Donnally were partners in the practice of dentistry in Cynthiana. Dr. Peckover's father a practising dentist at Paris, Ky., was also associated in some way with the firm. This threefold partnership was dissolved a month or two ago, and the settlements did not progress to the mutual content of the two younger members of the firm, and there was ill-feeling. On Wednesday, it is said, the two gentlemen, in a talk about the settlement, had a dispute over an account for $10 against a customer of the late firm. Donnally accused Peckover of cheating him. Peckover in reply to this assertion, denounced him as a liar. Dr. Donnally turned away with the remark that they would have no difficulty. Both of these gentlemen were men of sobriety and good standing in our community.

Meantime Donnally brooded over the wrong, and to a friend who was trying to dissuade him from taking any further notice of the insult, he said, in substance : "I have been thinking the thing over, and the more I think of it the more provoked I get." Thursday evening about 5.30 o'clock, Donnally stepped out of his office, with a four-inch Smith & Wesson pistol, and finding Peckover on the street, who had just emerged from his own office, which is adjoining that of Dr. Donnally, called to him. He turned around, and as he did so Donnally said: "You called me a liar yesterday," and the words had hardly fallen from his lips when he fired his pistol, the ball passing almost through Peckover, near the region of the heart. He got up and walked into a jewelry establishment, and said that "Charley Donnally had shot him," and died in about twenty minutes. Mr. James T. Hedges, a dry goods merchant, just opposite, stepped over and caught Donnally by the right shoulder and arm as he was in the act of shooting the second time, and told him he had killed Peckover, and should not shoot again. Constable Ewalt then came up and arrested Donnally. "My God doctor, what did you do that for?" exclaimed the officer. "I am as sorry for it as anybody," answered the prisoner. "Where do you want me to go?"

Deputy Marshal Hoffman joining the two, the whole party proceeded to the courthouse building and into the office of County Judge Lucius Desha, Jr. Judge Desha sat facing the only door and across the table from it, pouring over the statute law relating to the case in hand. Hoffman sat with his face at right angles to the door and near it, with his feet on the table. Ewalt was reared back in a chair close to the only window, his right side face to that window and his back to the door. Donnally sat reared back in his chair, his left face to the window.

R. H. Ridgeley, a brother-in-law of Peckover, was at the latter's house, his own home, when the news came of his kinsman's fate. He had been out only a few moments before, but was not well and returned to the house. What he did in the twenty minutes or so intervening between the death of Peckover and the shooting of Donnally can only be conjectured from the result. He got a pistol, a full sized Colt's army revolver. It is certain too that he said he was going to kill Donnally, but one young man to whom he said it thought it only an idle threat; and another gentleman with whom he talked on the subject left him believing or hoping that he had been dissuaded from his bloody design. He entered the court house yard by the gate, nearly opposite to, and a considerable distance from, the windows at which Ewalt and Donnally sat, passed by the window, around the corner of the house, and in at the door. The doomed man sat as described above, and the constable says his eyes were cast down, his feet on the front rounds of his chair. Hoffman saw Ridgely enter, pistol presented, and almost as quick as thought had his feet down from the table to the floor, himself on his feet, and his hand on Ridgeley's right arm. Neither Judge Desha nor Ewalt saw Ridgely enter, nor, probably did Donnally. Hoffman was too late. He thinks his striking Ridgely's arm lowered the course of the ball, and his idea agrees with the probability that the aim was at the head or the heart. Ridgley advanced about three or four feet into the room toward Donnally, and when four or five feet distant from him fired, saying as he fired, "Blood for blood." Some statements represent him as saying also, "I didn't do you as you did him. I came up to your face," and "I could have shot you through the window." He was seized, disarmed, and after a little delay, lodged in jail.

Donnally fell over from his chair to the floor, cried out in agony "My God," asked Judge Desha to tell his brother to take care of his effects and attend to everything; the death pallor overspread his face, and he expired in a few minutes. His body was removed to his office room.

Coroner Whitaker held an inquest on the two bodies and simple verdict of death from pistol shots in the hands of C. L. Donnally and R. H. Ridgeley, respectively, rendered.

Dr. Peckover was about thirty-eight years old, weighing probably one hundred and sixty-five pounds, rather heavy-set and in excellent health. He was in the quartermaster's office in Nicholasville, Ky., during the war; afterwards lived in Louisville a year or two and established himself as a dentist in Cynthiana some eight or nine years ago. He married a daughter of R. H. Ridgeley, in Nicholasville.

Dr. Donnally was a Virginian, had been in Cynthiana for several years, and acquired the repute of a genial gentleman as well as a highly skilled practitioner of his profession. He leaves no family, never having been married. He was about forty-two years old, rather taller than Peckover, and about the same weight.

Ridgley is a young man, bearing a blameless reputation, and is described as a rare exception to the generality of young men. His attachment to his father and brother-in-law was very tender, and these circumstances, with the feeling that Donnally was utterly wrong in his attack on Peckover, and profound sympathy for Peckover's family, make it entirely certain that the current of opinion in his favor will make his conviction impossible.

---

### MONTHLY SUMMARY.

